# Prevalence and Morphology of C-Shaped Canals in Mandibular Second Molars: A Cross-Sectional Cone Beam Computed Tomography Study in an Ecuadorian Population

**DOI:** 10.3390/dj13040147

**Published:** 2025-03-28

**Authors:** Josué Fernández Laplace, Jenny Guerrero Ferreccio, Giulia Malvicini, Catalina Mendez de la Espriella, Alejandro R. Pérez

**Affiliations:** 1 Graduate Endodontics Program, School of Dentistry, Universidad Catolica de Santiago de Guayaquil (UCSG), Guayaquil 090615, Ecuador; josue.fernandez@cu.ucsg.edu.ec (J.F.L.); jenny.guerrero01@cu.ucsg.edu.ec (J.G.F.); 2 School of Dentistry, Universidad Catolica de Santiago de Guayaquil (UCSG), Guayaquil 090615, Ecuador; 3 Unit of Endodontics and Restorative Dentistry, Department of Medical Biotechnologies, University of Siena, 53100 Siena, Italy; giulia.malvicini@student.unisi.it; 4 Post Graduate Endodontic Program, Department of Endodontics, College of Dental Medicine, Nova Southeastern University, Fort Lauderdale, FL 33328-2018, USA; cmendez@nova.edu; 5 Department of Endodontics, Rey Juan Carlos University, 28922 Alcorcón, Madrid, Spain; 6 Surpreendente Research Group, 4400-239 Vila Nova de Gaia, Portugal

**Keywords:** anatomy, cone beam computed tomography, C-shaped canal, dental pulp cavity

## Abstract

**Background/Objectives**: C-shaped canals represent a challenge in endodontic procedures. The present study aimed to investigate the prevalence and characteristics of C-shaped canals in mandibular second molars in Guayaquil, Ecuador. **Methods**: CBCT records from two radiology centers were examined (2020–2022). A total of 400 CBCT scans (800 mandibular second molars) were analyzed using Fan’s classification. Data on the presence or absence of C-shaped canals, gender, and bilateral occurrence were collected. Statistical analysis included the test for differences in proportions and the chi-squared test to assess the significance of correlations between variables. **Results**: C-shaped canals were found in 28% of mandibular second molars, affecting 33.75% of the patient sample. The prevalence was significantly higher in females (42.9%) compared to males (20.4%). Bilateral occurrences were observed in 63% of affected patients. Morphologic variability was pronounced, with Fan C1 and C4 dominating in the coronal and apical thirds, respectively, while a shift toward Fan C2 and C3 was observed in the middle third. Only 19.2% of the teeth maintained a consistent morphology. **Conclusions**: C-shaped canals were found in 28% of mandibular second molars and occurred predominantly in females. Notably, most cases were bilateral, highlighting the importance of effectively adapting endodontic techniques to treat this unique anatomical variation. Further research into genetic and environmental influences could deepen our understanding and help develop refined diagnostic and therapeutic strategies.

## 1. Introduction

Successful endodontic treatment requires a comprehensive understanding of root canal morphology, as anatomical variations can significantly affect treatment planning and outcome. Recognizing these variations, particularly those prevalent in different ethnic groups, enhances the clinician’s ability to treat pulpal and periapical pathologies predictably [1,2,3,4,5].

One of the most complex and challenging anatomical variations encountered in endodontics is the “C-shaped canal” configuration, a characteristic morphology that resembles the letter “C” in cross-sections [3,6]. This variation is found primarily in mandibular second molars and often has an irregular internal anatomy, including deep pulp chambers, isthmuses, and complex canal bifurcations. The crescentic structure of the C-shaped canal system may extend as a continuous ribbon or have anastomoses that create unpredictable pathways within the root and make cleaning and obturation of the canal difficult [3,6]. The C-shaped canal configuration was first documented by Cooke and Cox in the 1970s, highlighting its significant clinical implications in endodontics [7,8].

The etiology of the C-shaped canals is primarily attributed to a developmental anomaly in which the epithelial root sheath of Hertwig does not fuse completely on either the lingual or buccal side of the molar root [9]. Another theory suggests that these canals are formed by contiguous cementum deposition along the root [3,10]. Although genetic factors are suspected, the exact genetic determinants have not yet been identified.

From a clinical point of view, the C-shaped canal represents a significant challenge for endodontic treatment [11]. The complex anatomy of these canals makes cleaning, shaping, and obturation difficult, as the deep pulp chambers, isthmuses, and connecting accessory canals make thorough debridement difficult [12]. Because of this complexity, the American Association of Endodontics (AAE) has classified C-shaped canals as highly complex cases. It advocates using specialized techniques and imaging, particularly three-dimensional imaging such as cone beam computed tomography (CBCT), to aid diagnosis and treatment planning [13,14,15,16].

Although the prevalence of C-shaped canals has been extensively studied worldwide, there is still little data on their prevalence in Latin American populations. Regional differences in the prevalence of these canals highlight the importance of conducting localized studies to better inform clinical and academic practice. For example, some Asian populations report prevalence rates as high as 44%, while in Western countries such as Portugal, Spain, and the United States rates typically range from 6 to 14% [2,3,4,10,11,17]. This study aims to fill this gap by providing data on the prevalence and morphology of C-shaped canals in the Ecuadorian population, thus contributing to a broader understanding of anatomical variations in endodontics.

This study investigates the prevalence, anatomical features, and clinical implications of C-shaped canals in mandibular second molars in an Ecuadorian sample. Analyzing the frequency and patterns of these configurations aims to inform clinical endodontic strategies and improve global knowledge of C-shaped canal morphology in different populations.

## 2. Materials and Methods

### 2.1. Sample Size

The sample size of this study was carefully calculated using Epidat software (v3.1, Conselleria de Sanidade de la Xunta de Galicia, Santiago de Compostela, Spain) to represent the adult population of Guayaquil, Ecuador, estimated at 2,698,000. Considering a margin of error of 5%, a confidence level of 95%, and an assumed heterogeneity of 50%, the required sample was set at 385 individuals. To achieve the sample size of the only known worldwide prevalence study to date, the sample was adjusted to 400 CBCT scans.

### 2.2. Data Compilation

The present study was approved by the Research and Ethics Committee of the Faculty of Dentistry, Pontificia Universidad Javeriana, and received the registration number OD-0285. The data were compiled from the databases of two radiology centers in Guayaquil, covering the period from 2020 to 2022. At baseline, 1580 CBCT scans from 1580 patients were included. A single operator evaluated all scans, following a standardized protocol to ensure consistency. Four hundred scans met the predefined inclusion and exclusion criteria and were selected for this study.

### 2.3. Inclusion and Exclusion Criteria

The inclusion criteria for this study were as follows:

- CBCT scans show the entire mandibular dental arch.
- Mandibular second molars with fully developed roots are present.
- The mandibular second molars are present on both sides.
- A minimum layer thickness of 0.6 mm.

The exclusion criteria were as follows:

- Incomplete root development of the molars.
- Insufficient field of view to exclude the examination of both hemiarches.
- The presence of root canals with artifacts interferes with the evaluation of the axial slices.
- Poor-quality CBCT scans compromise the visualization of the internal anatomy.
- Endodontically treated mandibular second molars.

### 2.4. Image Acquisition

CBCT scans were previously acquired using a Kodak 9000 3D system (Kodak Carestream Health, Trophy, France) with an exposure protocol set at 70 kV, 10 mA, and 10.8 s. The scans were performed using a 50 × 37 mm field of view (FOV) and a 76 μm isotropic voxel size to ensure high-resolution imaging.

### 2.5. Tomographic Analysis

The tomographic analysis was conducted on an ASUS ROG laptop with a 17 inches FHD display (ASUSTeK Computer Inc., Beitou District, Taipei, Taiwan). One operator experienced in CBCT interpretation evaluated the images using a specialized software (One Volume Viewer v2.6.0 and NNT Viewer v8.0) provided by the CBCT machine manufacturers. The assessment was performed on a 1920 × 1200-pixel LCD monitor under standardized conditions in a silent, dimmed-light room to minimize distractions and optimize visualization.

A structured step-by-step methodology ensured consistency across all samples. CBCT scans focused on axial sections of the mandibular second molars at three predefined levels: the coronal, middle, and apical thirds. The coronal level was defined as 2 mm below the cemento–enamel junction, the apical level as 2 mm above the anatomical apex, and the middle level as the midpoint between them.

**Figure 1:**
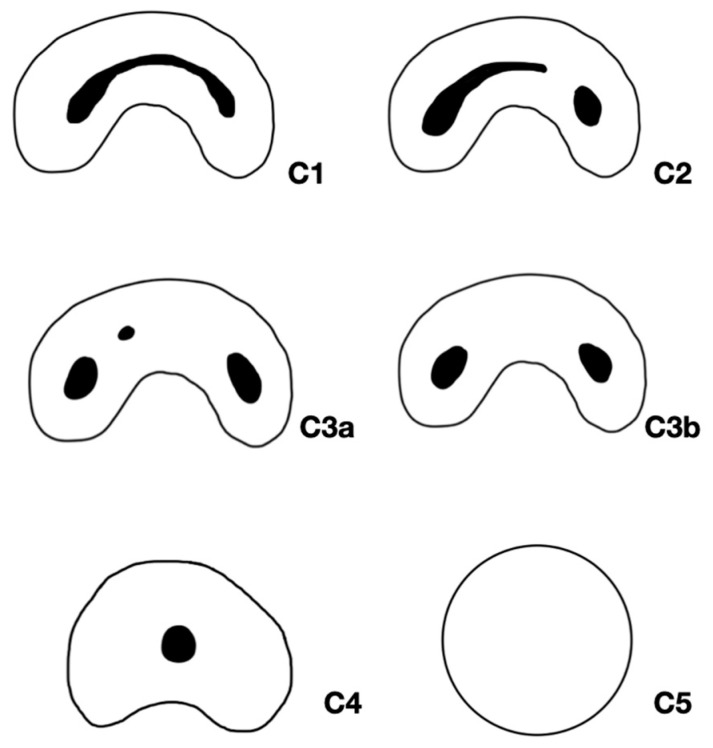
Graphical representations of the C-shaped canal configuration according to Fan et al. [13]. C1 shows a continuous “C”-shaped canal; C2, a semicolon-shaped morphology; and C3, three or two separated canals (illustrated as C3a and C3b). C4 represents a single, round or oval canal, while C5 indicates the absence of a visible canal lumen.

**Figure 2:**
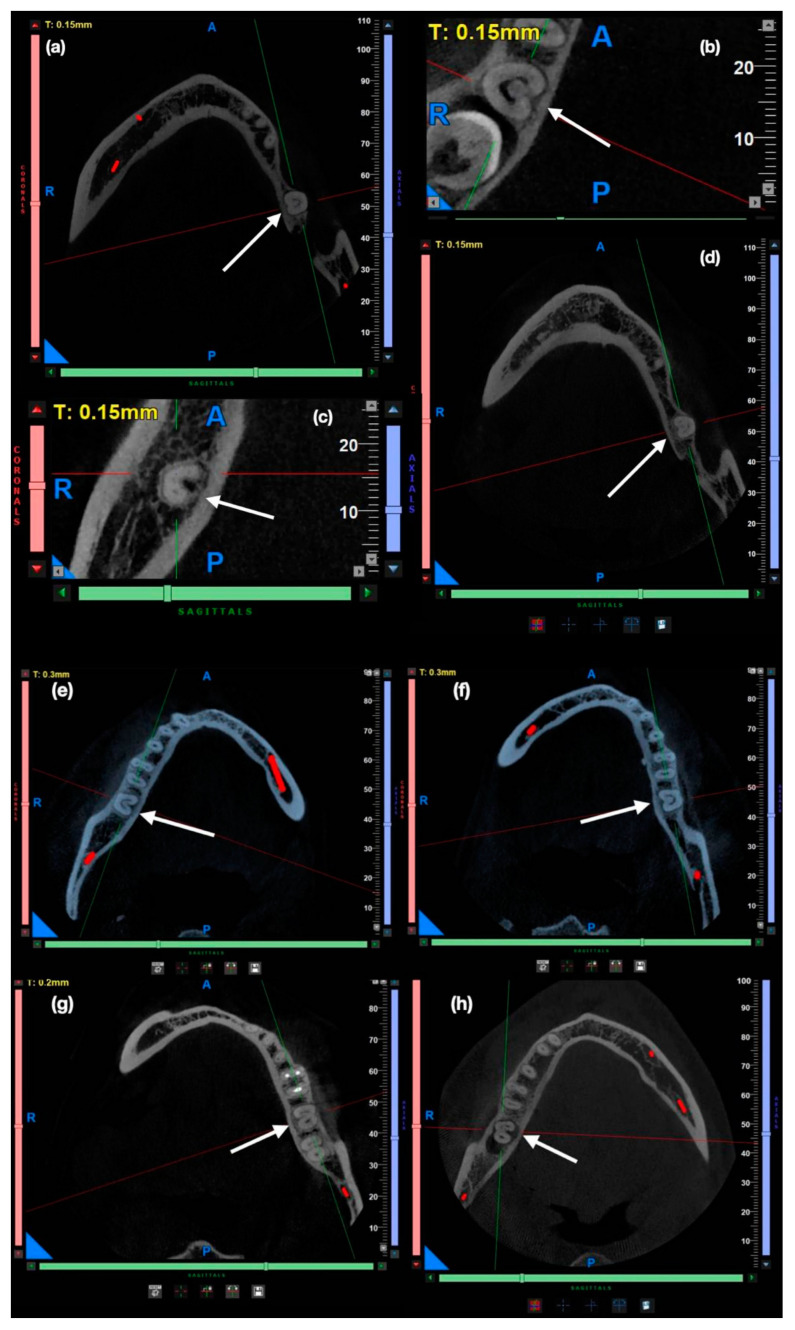
CBCT axial slices illustrating variations in the morphology of C-shaped canals, highlighting elongation, curvature, and potential continuity. Images (**a**–**d**,**h**) were reconstructed with slice thicknesses of 0.15 mm, (**e**,**f**) 0.3 mm, and (**g**) 0.2 mm using post-processing software for enhanced visualization. These reconstructed slice thicknesses differ from the anatomical wall thickness measurements used in this study and were selected solely for illustrative purposes. White arrows indicate teeth with C-shaped canals.

The classification of C-shaped canals followed the methodology described by Fan et al. [13] (Figure 1), which categorizes them into five types: C1 (continuous “C” shape), C2 (semi-columnar morphology), C3 (two or three discrete canals), C4 (a single round or oval canal), and C5 (absence of a visible canal lumen). Figure 2 illustrates representative CBCT axial slices depicting these morphological variations, providing a visual reference for canal assessment in this study.

### 2.6. Variables

The following variables were collected in this study:

- C-shaped canals: the primary dependent variable, scored as either present or absent, with absolute frequency and percentage used for analysis.
- Sex: a dichotomous nominal variable indicating biological sex.
- Root canal anatomy: analyzed using the Fan classification, which has five categories.
- Bilaterality: categorically scored as yes/no.
- Root wall thickness: measured at different levels (coronal, middle, and apical thirds) along the entire length of the C-shaped canals in teeth 37 and 47.

### 2.7. Root Canal Wall Thickness Measurement

Root canal wall thickness was assessed at three standardized levels—coronal (1 mm below the canal orifices), middle (midpoint between the coronal and apical thirds), and apical (3 mm from the root apex)—in mandibular second molars (teeth 37 and 47) using axial CBCT images. Measurements were performed with calibrated digital tools in NNT Viewer (NewTom, Verona, Italy) and One Volume Viewer (Carestream Dental, Atlanta, GA, USA). At each level, the shortest distance from the internal canal wall to the external root surface was recorded. Each measurement was repeated three times by a calibrated operator, and the mean value was used for analysis. This approach enabled consistent and reproducible evaluation of wall thickness across root thirds and between contralateral teeth.

### 2.8. Statistical Analysis

The data were systematically processed in an automated database using STATA BE (version 18, StataCorp LP, College Station, TX, USA).

Continuous variables were regarded as the mean and standard deviation (SD), while binomial or categorical variables were regarded as the number of observations (N) and proportions (percentage—%). Verification of the normal distribution of data was assessed using the Shapiro–Wilk test. Continuous variables were compared using the Kruskal–Wallis test, followed by the Dunn test for post hoc comparison, while binary and categorical variables were compared using the chi-squared test. The significance level was set at 95% (*p* < 0.05). The results are presented in frequency distribution tables and graphs to provide a clear and comprehensive understanding.

## 3. Results

### 3.1. Epidemiological Characteristics

This study analyzed 800 mandibular second molars from 400 patients (400 right molars and 400 left molars). The sample comprised 238 females (59.5%) and 162 males (40.5%), with an average patient age of 39.5 years (SD ± 14.38). While the gender distribution (Table 1) was statistically significant (X^2^ = 13.19, *p* = 0.00), this finding reflects only the demographic composition of the sample and does not indicate a gender-based prevalence of C-shaped canals. These data only indicate more women among the patients in the sample.

**Figure 3:**
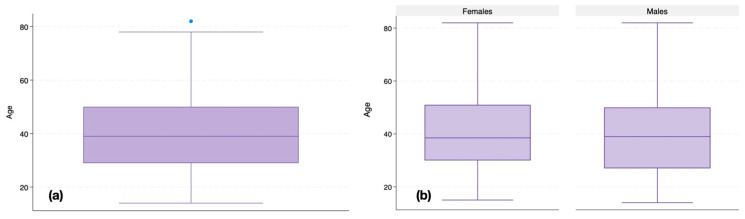
Box plots showing age distribution (**a**) overall and (**b**) categorized by gender. The blue dot represents an outlier value, while the dashed lines indicate the interquartile range and whiskers of the box plot.

Figure 3 illustrates the age distribution of the samples both overall (Figure 3a) and stratified by gender (Figure 3b). The mean age for females was 39.94 years (SD ± 14.12, range: 15–82), while for males it was 38.93 years (SD ± 14.78, range: 14–82), with an interquartile range (IQR) spanning from around 30 to 50 years. In this dataset, the minimum observed ages were 15 years for females and 14 years for males, while the maximum recorded age was 82 years for both groups. The presence of outliers above 80 years indicates a few individuals whose ages deviate significantly from the main distribution.

However, no statistically significant differences in age distribution were observed between males and females. These data confirm that age was evenly distributed across genders, reducing the likelihood of age-related bias in this study.

### 3.2. Prevalence of C-Shaped Canals in Mandibular Second Molars

Of the 800 mandibular second molars examined, 224 teeth (28.0%) exhibited C-shaped canals, affecting 135 patients, 33.75% of the total patient sample.

When analyzed by gender, the prevalence was significantly higher in females (42.9%) compared to males (20.4%). This distribution was also statistically significant (*p* = 0.00; Table 2).

### 3.3. Bilaterality of C-Shaped Canals

Of the 135 patients with C-shaped canals, 85 individuals (63%) had a bilateral presence (both left and right mandibular second molars), while the remaining 50 patients (37%) had unilateral C-shaped canals. This bilaterality was statistically significant (X^2^ = 7.54, *p* = 0.006), demonstrating a higher tendency for bilateral C-shaped canals in patients.

### 3.4. Fan Classification by Root Thirds

The results regarding the root canal anatomy are shown in Table 3.

Among the 224 mandibular second molars with C-shaped canals, 42 teeth (19.2%) exhibited a consistent Fan classification across all root thirds, while the remaining 182 teeth (81.25%) demonstrated anatomical variation along the root length.

The distribution of Fan classifications overall was analyzed across the coronal, middle, and apical thirds of the root. In the coronal third, the most common configurations were Fan C1 (50.45%) and Fan C4 (23.21%), while Fan C2 (12.92%) and Fan C3 (13.39%) were less frequently observed. No significant differences were found between the left and right mandibular second molars (X^2^ = 0.30, *p* = 0.96).

In the middle third, Fan C2 (34.82%) was the most prevalent classification, followed by Fan C1 (26.79%), Fan C3 (26.34%), and Fan C4 (12.05%). Similar to the coronal third, there were no statistically significant differences between the left and right mandibular second molars (X^2^ = 3.39, *p* = 0.34).

The apical third demonstrated the greatest morphologic variation, with Fan C4 (43.75%) being the most commonly observed pattern, followed by Fan C1 (26.79%), Fan C3 (24.11%), and Fan C2 (5.8%). As with the other root sections, no statistically significant differences were found in the distribution of Fan classifications between tooth 37 and tooth 47 (X^2^ = 0.31, *p* = 0.96).

Furthermore, the distribution of Fan classifications was analyzed separately for uniform and non-uniform C-shaped mandibular second molars at the coronal, middle, and apical levels to determine if significant differences exist between the left (tooth 37) and right (tooth 47) second molars.

Within the uniform group, C4 was the most prevalent classification (24 teeth, 57.14%), followed by C1 (13 teeth, 30.95%), C3 (4 teeth, 9.52%), and C2 (1 tooth, 2.38%).

In contrast, the 182 non-uniform teeth displayed changes in Fan classification at different root thirds. At the coronal-to-middle-third transition, C1 frequently shifted to C2 (43 cases, 23.6%) and C3 (29 cases, 15.9%), while C4 remained broadly stable.

Moving from the middle to the apical third, C1 transitioned predominantly to C4 (28 cases, 15.4%), while C2 and C3 displayed high variability, shifting frequently to C1 or C4.

Finally, at the apical transition, C4 showed the greatest instability, with 29 cases (15.9%) shifting to C1, whereas C1 and C3 remained relatively more stable.

Table 4 presents the distribution of transition patterns across different root thirds, highlighting the variability in root canal morphology.

Among uniform teeth (n = 42), the distribution of Fan classifications did not significantly differ between the right and left second mandibular molars at any root level (coronal: X^2^ = 1.2644, *p* = 0.738; middle: X^2^ = 1.2644, *p* = 0.738; apical: X^2^ = 1.2644, *p* = 0.738).

Similarly, among non-uniform teeth (n = 182) no statistically significant differences were observed between the right and left second mandibular molars in the distribution of Fan classifications at any level (coronal: X^2^ = 2.13, *p* = 0.54; middle: X^2^ = 3.17, *p* = 0.37; apical: X^2^ = 0.02, *p* = 0.99).

These results indicate that the distribution of Fan classification patterns is consistent across the left and right mandibular second molars, regardless of whether the C-shaped canal morphology is uniform or non-uniform.

### 3.5. Root Canal Wall Thickness

Intragroup and intergroup comparisons of wall thickness were performed for teeth 37 and 47, as shown in Table 5. No significant differences were found between the right and left mandibular second molars across the entire root length (coronal, middle, and apical thirds). However, within each group the wall thickness varied significantly among the coronal, middle, and apical thirds (*p* < 0.001).

## 4. Discussion

This study investigates the epidemiologic and anatomic features of C-shaped canals in mandibular second molars, focusing on their prevalence, bilaterality, and morphologic variability between root thirds. The results highlight the clinical challenges of this complex root canal configuration and point to demographic factors that may influence its occurrence.

The gender distribution in this study—42.9% women and 20.4% men—is statistically significant and indicates a higher prevalence of C-shaped canals in women in this population (Table 1). Although gender has been discussed as a variable while studying C-shaped canals [18], it is generally less emphasized than factors such as ethnicity and geographic region [6]. Some studies have documented that females are more likely to have C-shaped canal configurations in mandibular second molars, possibly due to genetic predispositions that influence root development [11,17,18]. However, further studies with a balanced gender representation are needed to confirm this trend in different populations [6]. This finding emphasizes that clinicians may need to consider gender as a factor in diagnosing mandibular molar root canal morphology.

In the present study, C-shaped canals were observed in 28% of the mandibular second molars examined, consistent with existing literature documenting a higher incidence in East Asian populations, where prevalence rates can be as high as 40% [6]. In contrast, studies in Western populations often report lower rates, between 2% and 11% [2,6]. This variation suggests that genetic and regional factors may influence the morphology of the C-shaped canal, although the specific mechanisms are still unclear. The prevalence rate identified here emphasizes the importance of regional studies in determining population-specific endodontic considerations, which may be critical for practitioners in different settings.

Among the 135 patients with C-shaped canals, 63% were bilateral. This finding is consistent with other reports suggesting a tendency toward symmetry in the morphology of C-shaped canals, with a C-shaped canal in a mandibular molar often predicting a similar configuration in the contralateral molar [2,3,9,19]. This bilateral tendency emphasizes the genetic basis of the morphology, which is probably due to developmental anomalies in Hertwig’s epithelial root sheath fusion [9]. Clinically, identifying a C-shaped canal on one side may indicate the configuration in the contralateral molar and allow for more targeted diagnostic imaging and treatment planning.

Nevertheless, unilateral occurrences were also observed (37%), suggesting that a genetic predisposition alone cannot fully explain this anatomical feature. Other factors may contribute to these asymmetrical patterns, including epigenetic influences that modulate gene expression during root development, biomechanical variations within the jaw that affect localized root morphology, and inherent embryological asymmetries that arise during early development. Additionally, differences in vascularization during odontogenesis or random developmental errors in the fusion of Hertwig’s epithelial root sheath could produce these unilateral anomalies [20,21,22].

This study classified C-shaped canals according to Fan’s system, documenting significant morphological variability across the root thirds. Forty-two teeth (19.2%) exhibited a consistent Fan classification across all root thirds, while the remaining 182 teeth (81.25%) demonstrated anatomical variation along the root length.

In the coronal third, the most common configurations were Fan C1 (50.45%) and Fan C4 (23.21%), while the middle third saw a shift, with Fan C2 (34.82%) being the most prevalent, closely followed by C1 (26.79%) and C3 (26.34%). The apical third showed the greatest morphologic variability, with Fan C4 (43.75%) predominating.

As previously demonstrated, C-shaped canal anatomies are not constant over the entire root canal length and can vary considerably [23]. This study analyzed the distribution of Fan classifications in two groups: one where the C-shaped anatomy was constant along the entire root length, and another where transitions between different canal shapes were present.

For the non-uniform group, the most prevalent transition patterns were calculated, revealing substantial morphological variability. The most frequent transition patterns in the dataset involve C1 (a continuous C-shaped canal) shifting into more complex configurations (i.e., C2, C3, or C4). This pattern suggests that while many mandibular second molars start with a relatively simple and continuous canal morphology (C1), structural modifications occur toward the apex.

This variability reflects the challenge of treating C-shaped canals, as these morphological shifts make cleaning and obturation difficult [24]. In particular, the high prevalence of Fan C4 in the apical third may pose a risk for incomplete debridement, as narrow and irregular areas tend to retain debris and harbor bacteria [25,26].

These findings are consistent with studies using micro-computed tomography and CBCT, emphasizing the complexity of C-shaped canals and their propensity for variable cross-sectional shapes along the root [6,13,15,27,28]. In clinical practice, this variability requires advanced imaging methods, as standard radiographs may not capture the full extent of the morphology, especially in the apical third [28].

This study demonstrates significant variations in wall thickness in C-shaped mandibular second molars, with a progressive reduction from the coronal to the apical third. Although no significant differences were observed between the right (tooth 47) and left (tooth 37) mandibular second molars, the thickness changes significantly within each root (*p* < 0.00).

The observed wall thinning in the middle and apical thirds is clinically relevant, as thinner dentinal walls increase the risk of iatrogenic errors such as stripping perforations [24]. The findings from the present study on root canal wall thickness variations in C-shaped mandibular second molars align with those observed by Martin et al. [24], who conducted a CBCT and micro-CT study to assess dentine thickness before and after instrumentation. In both studies, dentine thickness varies significantly within C-shaped mandibular second molars. However, while our study observed the apical third as the most vulnerable region, their findings suggest that middle and apical thirds both present structural risks, depending on the root canal type [24].

The clinical management of C-shaped canals is a significant challenge, mainly due to their irregular shape and the anatomical variations that occur along the root length. This study supports the need for clinicians to use three-dimensional imaging techniques such as CBCT to assess the canal configuration accurately before treatment. Given the high prevalence and complexity of the observed C-shaped canals, endodontic procedures should emphasize careful cleaning and shaping techniques with careful consideration of the morphological differences of each root third [25]. This anatomical complex may require several modifications to the traditional endodontic treatment protocol. The floor of the pulp chamber may differ from the usual configuration of two mesial and one distal canal orifices and, therefore, may require customized preparation of the access cavity, root canal debridement strategies, and obturation procedures. The ability to anticipate the presence of a C-shaped anatomy is indeed a clinical advantage [3].

However, this study has limitations. First, the sample is limited to a specific population group, which may affect the generalizability of the results to other demographic groups. Additionally, the evaluation was conducted by a single examiner, which, despite following a standardized protocol, may introduce observer bias and limit the reproducibility of the findings. Future studies should consider multiple evaluators to enhance reliability and minimize potential subjective interpretation.

Relying exclusively on Fan’s classification may overlook other nuanced variations within C-shaped canals, as other classification systems may provide complementary insights. Future studies should aim for a cross-population analysis with broader classification criteria to further elucidate the influence of demographic factors and canal morphology on endodontic outcomes.

## 5. Conclusions

The anatomical complexity of C-shaped canals presents significant technical challenges for effective debridement and obturation, particularly due to their morphological variability and reduced dentinal wall thickness in the apical regions. In this Ecuadorian population, the high prevalence and frequent bilateral occurrence of C-shaped canals further emphasize the need for clinicians to anticipate and adapt to these variations. Incorporating CBCT imaging into diagnostic and treatment protocols is essential for enhancing the predictability and success of endodontic procedures in such anatomically complex cases.

## Figures and Tables

**Table 1:** Prevalence of C-shaped morphology according to gender.

Sex	Frequency	%
Female	238	59.5
Male	162	40.5
Total	400	100

X^2^ = 13.19; *p* = 0.00.

**Table 2:** Distribution of cases with C-shaped canals according to sex.

C-Shaped						
Sex	Present		Absent		Total	
	N	%	N	%	N	%
Female	102	42.9	136	57.1	238	59.5
Male	33	20.4	129	79.6	162	40.5
Total	135	33.8	265	66.3	400	100

x^2^ = 20.8, *p* = 0.00. Abbreviations: N, number of observations.

**Table 3:** Fan classification distribution in different root thirds (coronal, middle, and apical) of tooth 37 and tooth 47. The table presents the frequency and percentage of each Fan category (C1, C2, C3, and C4) observed in the respective regions.

Tooth	Root Third	Fan C1	Fan C2	Fan C3	Fan C4
Tooth 37	Coronal	60 (52.17%)	15 (13.04%)	16 (13.91%)	24 (20.87%)
	Middle	31 (26.96%)	43 (37.39%)	25 (21.74%)	16 (13.92%)
	Apical	31 (26.96%)	7 (6.09%)	27 (23.48%)	50 (43.48%)
Tooth 47	Coronal	53 (48.62%)	14 (12.84%)	14 (12.84%)	28 (25.69%)
	Middle	29 (26.61%)	35 (32.11%)	34 (31.19)	11 (10.09%)
	Apical	28 (25.69%)	6 (5.50%)	27 (24.77%)	48 (44.04%)
Overall	Coronal	113 (50.45%)	29 (12.95%)	30 (13.39%)	52 (23.21%)
	Middle	60 (26.79%)	78 (34.82%)	59 (26.34%)	27 (12.05%)
	Apical	59 (26.34%)	13 (5.8%)	54 (24.11%)	98 (43.75%)

**Table 4:** Most prevalent transition patterns in teeth exhibiting changes in C-shaped anatomy, according to the classification proposed by Fan et al., across different root thirds.

Transition Pattern	Frequency	Percentage (%)
C1 → C1 → C1 → C2	1	0.55
C1 → C1 → C3 → C1	3	1.65
C1 → C1 → C4 → C1	9	4.95
C1 → C2 → C4 → C1	9	4.95
C1 → C2 → C4 → C1	6	3.30
C2 → C2 → C3 → C2	4	2.20
C3 → C3 → C4 → C1	4	2.20
C4 → C2 → C4 → C4	6	3.30

**Table 5:** Mean and standard deviation (SD) of the wall thickness measured at different levels (coronal, middle, and apical thirds) along the entire length of the C-shaped canals in teeth 37 and 47.

	Coronal (mm) Mean ± SD	Middle (mm) Mean ± SD	Apical (mm) Mean ± SD
**Tooth 37**	1.87 ± 0.5 ^A^	0.86 ± 0.42 ^B^	0.734 ± 0.41 ^C^
**Tooth 47**	1.86 ± 0.55 ^A^	0.92 ± 0.44 ^B^	0.70 ± 0.70 ^C^

^A, B, C^ Superscript letters indicate statistically significant differences. Different letters denote significant differences between groups. Same letters indicate no significant differences.

## Data Availability

The data supporting this study’s findings are available from the corresponding author upon reasonable request.
